# Comparison of Sugammadex and Neostigmine for Reversal of Vecuronium-Induced Neuromuscular Blockade in Pediatric Patients: A Prospective Randomized Study

**DOI:** 10.7759/cureus.116255

**Published:** 2026-09-15

**Authors:** Neha Tiwari, Aarti Agarwal, Shilpi Verma, Puneet Goyal, Nidhi Singh, Prabhaker Mishra, Sanjay Dhiraaj

**Affiliations:** 1 Anesthesiology and Intensive Care, Sanjay Gandhi Postgraduate Institute of Medical Sciences, Lucknow, IND; 2 Biostatistics and Health Informatics, Sanjay Gandhi Postgraduate Institute of Medical Sciences, Lucknow, IND

**Keywords:** neostigmine, neuromuscular blockade, pediatric anesthesia, residual blockade, sugammadex, tidal volume, train-of-four monitoring

## Abstract

Background and objective

Neuromuscular blocking agents (NMBAs) used during general anesthesia require effective reversal to prevent residual paralysis. Neostigmine, the conventional reversal agent, is associated with slower recovery and muscarinic side effects. Sugammadex selectively encapsulates amino-steroidal NMBAs, providing rapid and predictable reversal. This study compared the efficacy, safety, and recovery profile of sugammadex and neostigmine for reversal of vecuronium-induced neuromuscular blockade in children.

Methods

In this prospective, randomized, double-blind trial, 100 children aged two to 12 years (American Society of Anesthesiologists (ASA) I-III) undergoing elective surgery requiring general anesthesia were randomized to receive sugammadex (2 mg/kg) or neostigmine (50 mcg/kg) with glycopyrrolate (10 mcg/kg). Neuromuscular function was monitored using train-of-four (TOF) stimulation. The primary outcome was time to achieve TOF ratios of ≥0.9 and 1.0 after reversal. Secondary outcomes included residual neuromuscular blockade, functional respiratory recovery assessed by tidal volume, hemodynamic adverse events, and respiratory complications.

Results

Demographic and intraoperative characteristics were comparable between groups. The median time (±IQR) to achieve TOF ≥0.9 and 1.0 was significantly shorter with Sugammadex than with Neostigmine [70(65-108) vs 252(240-280) and 85.5(75-142) vs 272(255-304) seconds, respectively, p = 0.001]. Heart rate was significantly higher following reversal with neostigmine and glycopyrrolate than with sugammadex, with the difference peaking at five minutes and progressively narrowing thereafter, but remaining significant for up to 1.5 hours after arrival in the post-anesthesia care unit. Tidal volume increased more rapidly with sugammadex than neostigmine after reversal, although this exploratory secondary outcome does not establish a causal relationship.

Conclusions

Sugammadex provided significantly faster and more reliable reversal of vecuronium-induced neuromuscular blockade in children aged two to 12 years, compared to neostigmine (as shown by neuromuscular monitoring), with less tachycardia following reversal.

## Introduction

Neuromuscular blocking agents (NMBAs) facilitate endotracheal intubation and optimize surgical conditions during general anesthesia. However, inadequate reversal can result in residual neuromuscular blockade, leading to respiratory complications, delayed recovery, and prolonged hospital stay [1]. Neostigmine, the conventional reversal agent, acts indirectly by inhibiting acetylcholinesterase but exhibits variable efficacy, a ceiling effect, and muscarinic side effects, necessitating the concomitant administration of anticholinergics [2]. Sugammadex, a modified γ-cyclodextrin, selectively encapsulates amino-steroidal NMBAs, producing rapid, predictable, and complete reversal without muscarinic adverse effects [3]. While it is accepted to be safe and effective in adults [4], pediatric data from India remain limited, particularly for the reversal of vecuronium-induced neuromuscular blockade [5,6]. The present prospective study was undertaken to fill this gap, in which we compared the efficacy and safety of sugammadex and neostigmine in reversing vecuronium-induced neuromuscular blockade in children undergoing elective surgery under general anesthesia with endotracheal intubation.

## Materials and methods

This prospective, randomized, double-blind, controlled study was undertaken after approval from the Institutional Ethics Committee of Sanjay Gandhi Postgraduate Institute of Medical Sciences located in Lucknow, UP, India (approval no. PGI/BE/448/2022 dated 14 Nov 2022), and written informed consent was sought from the parents or legal guardians of all participants during the pre-anesthetic evaluation on the day before surgery. The trial was registered prospectively at the Clinical Trial Registry of India (no. CTRI/2023/02/050085 dated 26/02/2023). The period of enrollment of pediatric patients was from March 2023 to November 2024.

A total of 100 children aged two to 12 years, ASA physical status I-III, posted for elective surgery under general anesthesia with endotracheal intubation requiring vecuronium-induced neuromuscular blockade, were planned for inclusion. Patients with underlying neuromuscular disorders, myasthenia gravis, significant renal or hepatic dysfunction, known hypersensitivity to study drugs, anticipated difficult airway, or major intraoperative protocol deviations were excluded.

The patients were randomly allocated (1:1) using a computer-generated randomization sequence into the sugammadex group (group S) or neostigmine group (group N). Randomization was performed at the end of surgery, before planned extubation. The study was double-blinded. An anesthesiologist not involved in data collection prepared the study drug. The calculated dose of reversal agents was diluted to a uniform volume of 5 mL and administered intravenously over one minute in identical syringes to maintain blinding.

Anesthetic management of all patients was done by a standardized institutional protocol: routine fasting guidelines, induction with fentanyl (2 mcg/kg), propofol (2-3 mg/kg), and vecuronium (0.08-0.10 mg/kg) to facilitate tracheal intubation, and maintenance with sevoflurane in an oxygen-air mixture. A top-up dose of vecuronium (0.02 mg/kg) was administered whenever the second twitch (T2) reappeared on train-of-four (TOF) monitoring. Standard monitoring included electrocardiography, non-invasive blood pressure (upper arm, contralateral to the neuromuscular monitoring site), pulse oximetry, end-tidal carbon dioxide, concentration of inspired oxygen, end-tidal sevoflurane concentration, temperature, urine output, and quantitative neuromuscular monitoring.

Heart rate, systolic blood pressure (SBP), diastolic blood pressure (DBP), and tidal volume were recorded before administration of the reversal agent and at every minute for 15 minutes thereafter. Quantitative neuromuscular monitoring was performed using the TOF 3D acceleromyography (AMG) monitoring device (MIPM GmbH (Mammendorf, DEU) supplied in India by Baxter India Pvt. Ltd. (Gurugram, HR, IND)). Two pre-gelled surface electrodes were placed over the ulnar nerve at the ventral surface of the wrist, approximately 2 cm to 3 cm apart, and a piezoelectric transducer was positioned at the tip of the distal phalanx of the thumb to detect adductor pollicis muscle response. This hand was kept outside surgical drapes, accessible throughout the intraoperative period. The device’s internal automated calibration mode (auto mode) was utilized to establish the patient-specific supramaximal stimulation threshold. Following induction of anesthesia, TOF stimulation of the ulnar nerve was applied every 15 seconds, and the TOF ratio (amplitude of T4 divided by T1) was noted. Neuromuscular blockade was monitored quantitatively throughout intraoperatively and intermittently in the post-anesthesia care unit (PACU).

After surgery, sevoflurane was discontinued at the final surgical suture, and reversal was administered when T2 reappeared. Group S received sugammadex 2 mg/kg, while group N received neostigmine 50 mcg/kg with glycopyrrolate 10 mcg/kg. The child was extubated by an anesthesiologist blinded to group allocation. The criteria for extubation were spontaneous tidal volume of ≥5 mL/kg, facial grimacing, spontaneous limb movement, and a TOF ratio of 1.0. The interval from reappearance of T2 to attainment of a TOF ratio of 1.0 was measured, and it was synonymous with administration of the reversal agent to tracheal extubation. On arrival in the PACU, heart rate, BP, peripheral oxygen saturation (SpO₂), and the TOF ratio were recorded at 30 minutes, one hour, 1.5 hours, and two hours.

The primary outcome was the time from reversal administration to attainment of TOF ratios ≥0.9 and 1.0. Secondary outcomes included residual neuromuscular blockade, hemodynamic changes, assessment of respiratory muscle function recovery through tidal volume (measured from the anesthesia ventilator's integrated spirometry (flow sensor of the breathing circuit)), recurarisation, and adverse events, including bradycardia, tachycardia, hypotension, hypertension, bronchospasm, laryngospasm, hypersalivation, oxygen desaturation, postoperative nausea and vomiting, and allergic reactions. Recurarisation (recurrence of neuromuscular blockade) was defined as a sustained fall in TOF ratio below 0.9, confirmed on four consecutive measurements (spread over one minute), in a patient who had previously attained a TOF ratio of 1.0 and had been extubated, with or without accompanying clinical signs of respiratory insufficiency.

The sample size of this study was calculated from a previously published comparison of the two agents, in which the time to a TOF ratio of 1.0 was 2.30 ± 0.95 minutes with sugammadex and 14.50 ± 3.65 minutes with neostigmine, using an independent sample t-test (the calculated Cohen's d effect size of the mean difference was 4.57) [7]. As the obtained effect size was too large, a conservative effect size of 0.8 (≥0.8 is considered a large effect size) was assumed. With a two-sided significance level of 0.05 and 95% power of the study, 42 patients per group were required. Allowing about 20% for attrition and protocol violations, a sample size of 50 patients per group was decided. The sample size was calculated using software G*Power version 3.1.9.7 (Heinrich Heine University Düsseldorf, DEU).

Continuous variables were summarized as mean with standard deviation for normally distributed data or median with IQR, where the distribution was skewed. Normality of the data was tested using the Shapiro-Wilk test, with a threshold of p = 0.05. To compare the means/medians between two independent groups, the independent samples t-test/Mann Whitney U test was used, respectively, as appropriate. Categorical variables were summarized as numbers and percentages. To compare the proportions/test the association between the study groups and categorical variables, the chi-square test was used. In case the expected frequency was below five in any cell, Fisher's exact test was used. The one-way analysis of covariance (ANCOVA) test was used to compare the post-observation values between the two study groups after adjusting for the baseline value of the heart rate. A 'linear mixed-effects model' was used to evaluate changes from baseline during follow-up and to determine whether the pattern of change over time differed between sugammadex and neostigmine. The change from baseline was treated as the dependent variable, and drug, time, and the drug-by-time interaction were included as 'fixed effects,' and a 'subject-specific random intercept' was incorporated to account for the correlation arising from repeated measurements. A p-value <0.05 was considered statistically significant. Statistical analysis was performed using software SPSS Statistics version 27 (IBM Corp., Armonk, NY, USA).

## Results

In all, 118 children aged two to 12 years were assessed for the study. Finally, 100 were enrolled and analyzed, with 50 in each group, after excluding 18 children before randomization. The Consolidated Standards of Reporting Trials (CONSORT) [8] flow chart depicts enrollment, exclusions, and randomization of participants (Figure 1).

**Figure 1:**
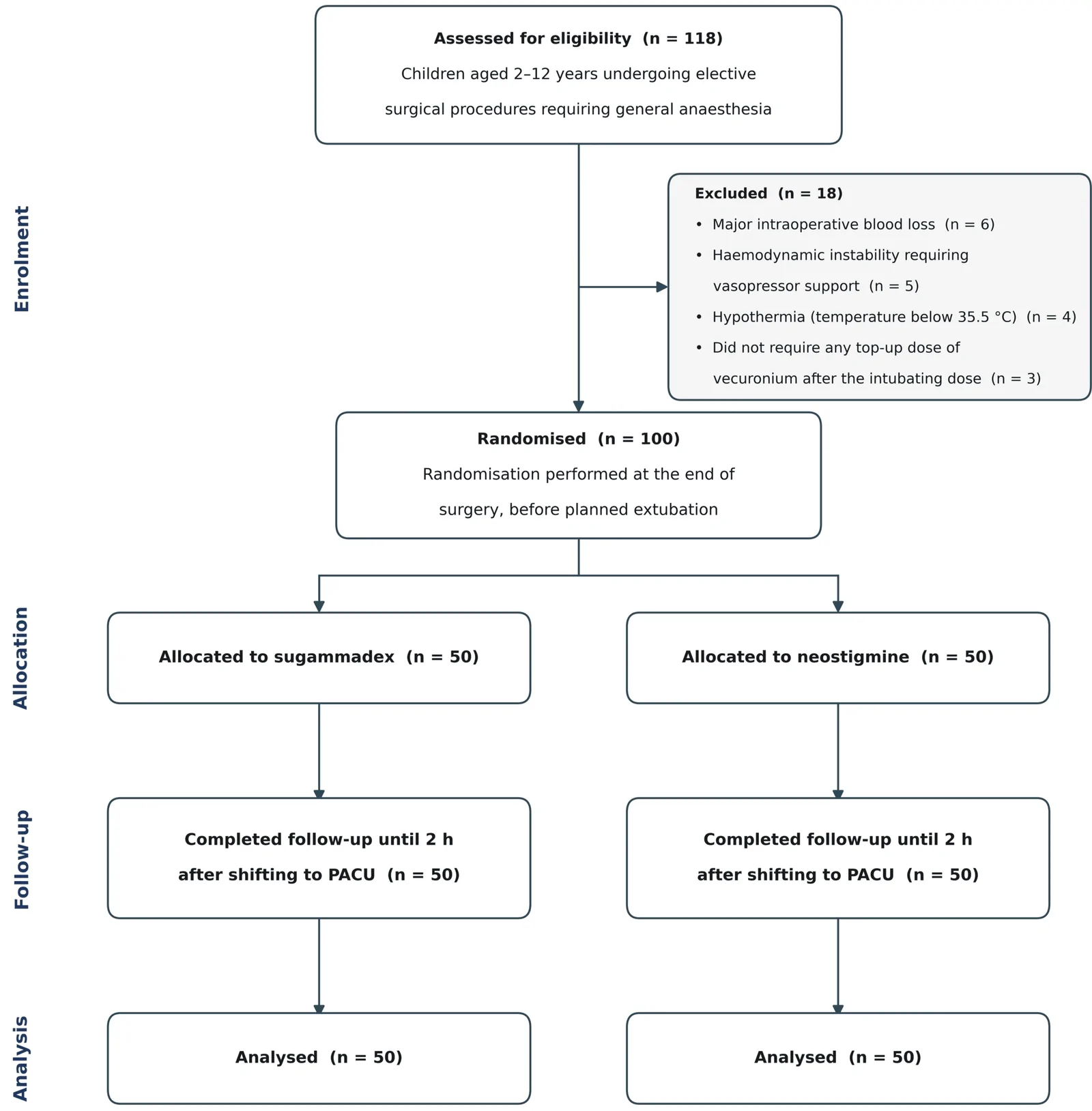
CONSORT flow chart illustrating enrollment, exclusion, and randomization of participants CONSORT: Consolidated Standards of Reporting Trials [8]; PACU: Post-anesthesia care unit

Baseline demographics, including age, sex, weight, height, BMI, and ASA physical status of children, were comparable among groups. The mean age was 6.21±3.15 years in the sugammadex group and 7.08±3.23 years in the neostigmine group. A male preponderance was found in both groups. The majority of children in both groups belonged to ASA physical status class I. The total dose of vecuronium administered was 0.31 ± 0.12 mg/kg in the sugammadex group and 0.28 ± 0.11 mg/kg in the neostigmine group. Duration of anesthesia was similar in both groups. The interval between the last vecuronium administration and reversal was comparable between groups (43.5 ± 6.8 vs 44.9 ± 7.1 minutes, p = 0.379). Reversal was administered in both groups at a standardized depth of neuromuscular blockade, defined by the reappearance of the T2 on quantitative monitoring (Table 1). Renal surgeries constituted the majority of procedures in both groups, followed by abdominal, plastic, and neurosurgical procedures.

**Table 1:** Demographic and clinical characteristics of children in both groups (total n =100) Data are presented in mean ± SD and compared using the independent samples t-test. Numbers and percentages are compared using the chi-square test/#Fisher's exact test. ASA: American Society of Anesthesiologists

Variable		Sugammadex group (n=50)	Neostigmine group (n=50)	Test statistics (T/chi square)	p-value
Age (years)		6.21±3.15	7.08±3.23	1.364	0.216
Age groups (years)	2-5	20 (40%)	18 (36%)	4.249	0.119
	6-8	18 (36%)	11 (22%)		
	9-12	12 (24%)	21 (42%)		
Sex	Male	43 (86%)	38 (76%)	1.624	0.308
	Female	7 (14%)	12 (24%)		
ASA physical status^#^	I	42 (84%)	41 (82%)	-	0.125
	II	5 (10%)	9 (18%)		
	III	3 (6%)	0		
Weight (kg)		20.99±9.90	22.97±9.87	1.002	0.250
Height (cm)		110.90±20.89	115.50±23.63	1.031	0.305
BMI (kg/m^2^)		16.29±3.30	16.52±1.64	0.441	0.202
Duration of anesthesia (hours)		4.47 ± 2.20	4.41 ± 1.63	0.155	0.856
Cumulative vecuronium dose	Total (mg)	5.90 ± 2.31	6.06 ± 2.69	0.319	0.456
	mg/kg	0.31 ± 0.12	0.28 ± 0.11	1.24	0.217
The relaxant-to-reversal interval (minutes)		43.5 ± 6.8	44.9 ± 7.1	1.007	0.379

Median time to a TOF ratio of 0.9 was 70 seconds with sugammadex versus 252 seconds with neostigmine, and total time to a ratio of 1.0 was 85.5 vs 272 seconds (Figure 2). The additional time required to recover from a TOF ratio of 0.9 to 1.0 was also shorter with sugammadex than neostigmine (15 vs 20 seconds, p=0.021). Although this difference was statistically significant, its magnitude was small relative to the much larger difference in overall time required to reach TOF 0.9. Recovery trajectory was entirely different in both groups, with the slowest sugammadex group pediatric patient reaching 1.0 at 204 seconds and the fastest neostigmine group pediatric patient at 180 seconds, and only a couple of patients overlapped. The rank-biserial correlation value of 0.996 indicated consistently faster recovery with sugammadex over neostigmine (Table 2).

**Figure 2:**
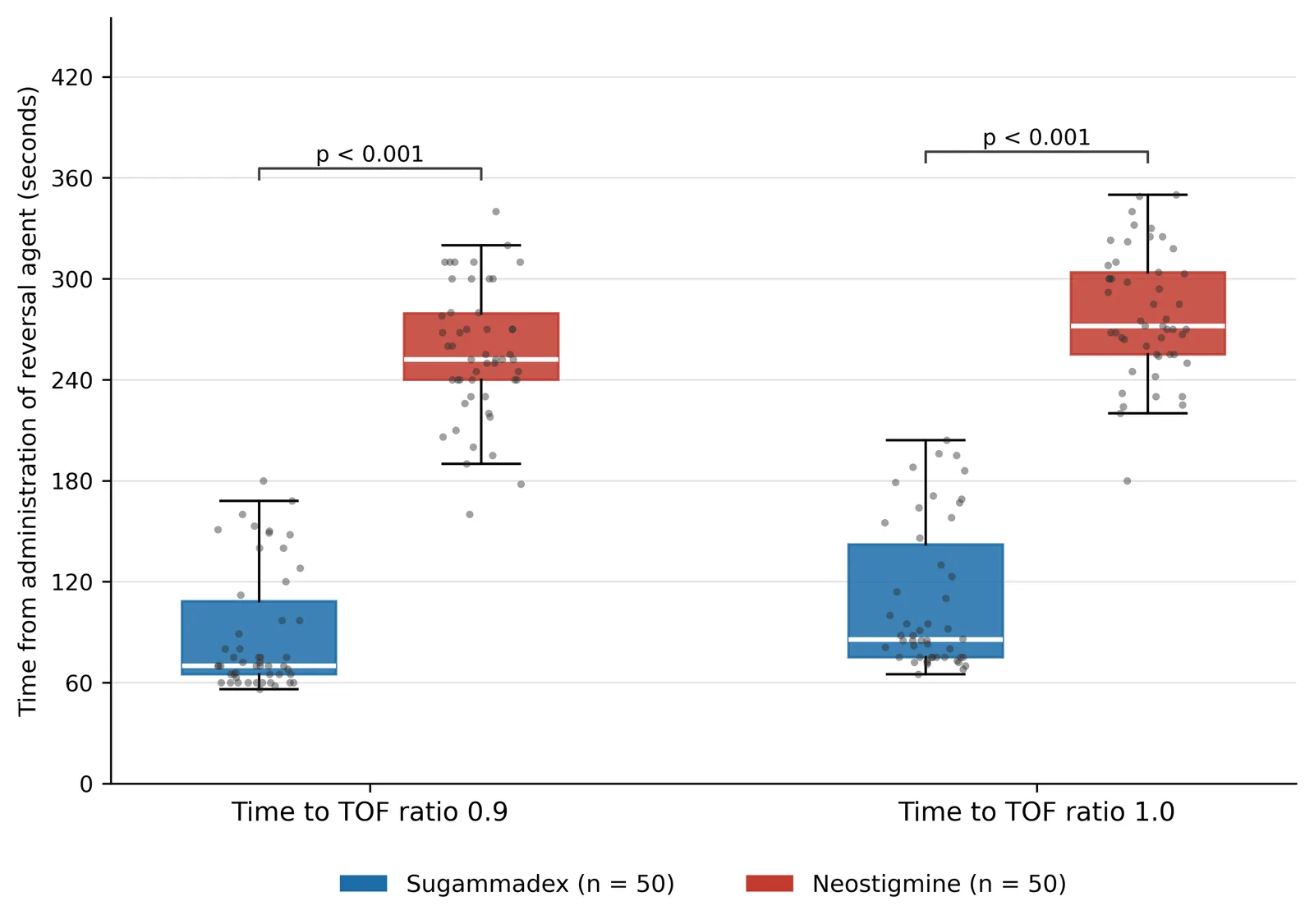
Time from administration of the reversal agent to a TOF ratio of 0.9 and 1.0 in pediatric patients receiving sugammadex or neostigmine Boxes show the median and IQR; whiskers extend to the most extreme value within 1.5 times the IQR of the box; n = 50 per group; p-values by Mann-Whitney U test. This graph was generated using R software version 4.5.1 (R Foundation for Statistical Computing, Vienna, AUT). TOF: Train-of-four

**Table 2:** TOF recovery characteristics in both groups (total n=100) Data are presented as median (first quartile-third quartile), compared using the Mann-Whitney U Test. A p-value <0.05 is considered significant. TOF: Train-of-four

Variables	Sugammadex median (IQR)	Neostigmine median (IQR)	Rank-biserial correlation	p-value
Time to TOF 0.9 (seconds)	70 (65-108)	252 (240-280)	0.997	< 0.001
Additional time, 0.9 → 1.0 (seconds)	15 (10-23)	20 (15-30)	0.267	0.021
Total time to TOF 1.0 (seconds)	85.5 (75-142)	272 (255-304)	0.996	< 0.001

Clinical criteria for extubation were already met in the majority of pediatric patients when a TOF ratio of 0.9 was reached. Extubation was nevertheless deferred until a ratio of 1.0 was attained. Because extubation was performed upon attainment of a TOF ratio of 1.0, the interval from administration of the reversal agent to tracheal extubation was the same as the time from T2 to a TOF ratio of 1.0.

Two pediatric patients in the neostigmine group required transient respiratory support after extubation (one required reintubation and one had to be given continuous positive airway pressure via face mask), but no such events occurred in the sugammadex group. This difference was neither statistically significant nor was the study powered to detect differences in infrequent respiratory events. Both episodes were recognized promptly, managed successfully, and the patients were discharged from the PACU with no further complications.

Following reversal, the neostigmine group demonstrated a progressive increase in heart rate over the subsequent five minutes, reaching a mean increase of 51% above baseline. In contrast, the sugammadex group showed only a modest transient rise in heart rate, peaking at approximately 12% at four minutes before returning towards baseline by five minutes. Five minutes after reversal, the mean heart rate was highest in the neostigmine group compared to the sugammadex group (153.6 ± 10.6 vs 120.4 ± 18.5 beats/min; mean difference, 33.2 beats/min; p < 0.001). The difference in heart rate was very large (Hedges' g = 2.19; 95% CI, 1.70-2.68), indicating a clinically substantial difference between the groups with minimal overlap of heart rate values. Subsequently, heart rate started slowing down, and the gap narrowed between the two groups, and by the time patients reached the recovery unit, the absolute rates had become similar (Figure 3).

**Figure 3:**
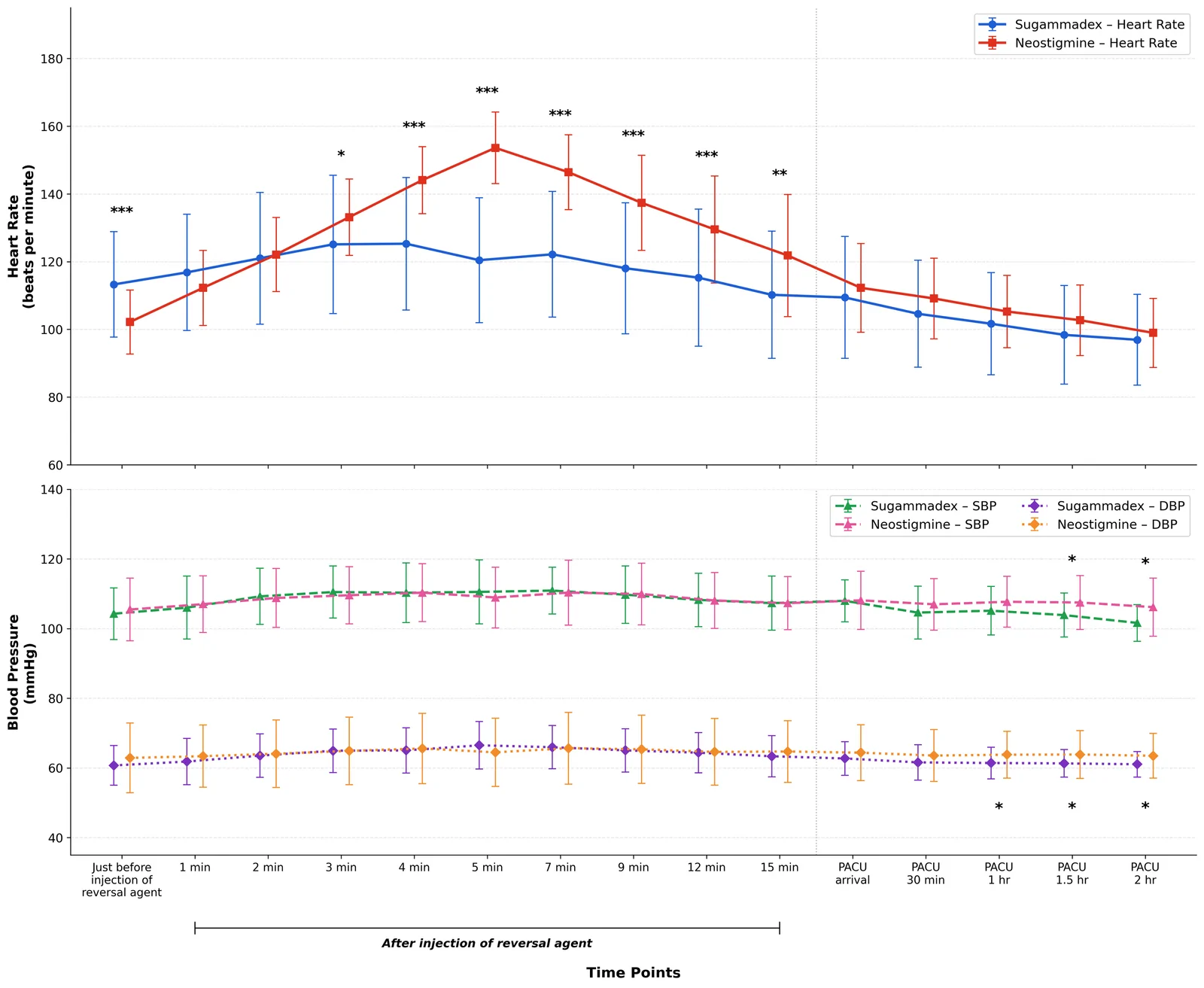
Heart rate (upper panel) and SBP and DBP (lower panel) in pediatric patients receiving sugammadex or neostigmine for reversal of vecuronium-induced neuromuscular blockade Values are presented as mean ± SD (n = 50 per group); Heart rate: *p<0.05, **p<0.01, ***p<0.001; Blood pressure: *p<0.05 (independent sample t-test). This graph was generated using R software version 4.5.1 (R Foundation for Statistical Computing, Vienna, AUT). SBP: Systolic blood pressure; DBP: Diastolic blood pressure

Since heart rate immediately before reversal was higher in the sugammadex group as compared to the neostigmine group (113.3 ± 15.6 vs 102.2 ± 9.5 beats/min, p < 0.001), between-group comparisons of subsequent heart rate were therefore adjusted for the pre-reversal value by analysis of covariance. After adjustment, heart rate was significantly higher in the neostigmine group at every time point from one minute after reversal until 1.5 hours in the recovery unit. Instead of attenuation, adjustment increased the observed difference, indicating that the unadjusted analysis underestimated the difference between the two groups (Table 3). The difference widened progressively to a maximum of 38.7 beats/min at five minutes after reversal (95% CI 32.8 to 44.6, p < 0.001), then narrowed, and was no longer significant at two hours (adjusted difference 5.4 beats/min, p = 0.073).

**Table 3:** ANCOVA between sugammadex and neostigmine group with differences adjusted for the pre-reversal value (total n=100) The adjusted difference is the estimated difference in heart rate in the group S minus group N for two pediatric patients with the same pre-reversal reading after adjusting the heart rate at baseline using the one-way ANCOVA. The F values were calculated with degrees of freedom of 1 and 97 throughout. A negative value means that heart rate was higher in the neostigmine group. A p<0.05 is considered significant. ANCOVA: Analysis of covariance ; PACU: Post anesthesia care unit; S: Sugammadex; N: Neostigmine

Time points	Unadjusted difference (group S − group N)	Adjusted difference (group S − group N)	95% confidence interval		Test statistics (F)	p-value
			Lower limit	Upper limit		
One minute after reversal	+4.58	-6.47	-9.35	-3.59	19.84	<0.001
Two minutes after reversal	-1.10	-11.19	-15.80	-6.58	23.20	<0.001
Three minutes after reversal	-8.02	-16.78	-22.43	-11.13	34.76	<0.001
Four minutes after reversal	-18.78	-24.99	-30.96	-19.02	69.03	<0.001
Five minutes after reversal	-33.18	-38.69	-44.61	-32.76	167.95	<0.001
Seven minutes after reversal	-24.24	-30.90	-36.62	-25.18	114.91	<0.001
Nine minutes after reversal	-19.32	-26.72	-33.05	-20.39	70.18	<0.001
12 minutes after reversal	-14.24	-21.45	-28.46	-14.43	36.84	<0.001
15 minutes after reversal	-11.60	-17.96	-25.31	-10.62	23.56	<0.001
On arrival in PACU	-2.84	-9.01	-15.12	-2.91	8.59	0.004
30 minutes in PACU	-4.52	-10.02	-15.44	-4.60	13.47	<0.001
One hour in PACU	-3.60	-9.53	-14.37	-4.70	15.34	<0.001
1.5 hours in PACU	-4.32	-9.86	-14.60	-5.12	17.02	<0.001
Two hours in PACU	-0.30	-5.35	-11.22	+0.51	3.28	0.073

At extubation, there was a 5% to 9% rise in SBP and DBP in both groups. It was statistically indistinguishable until one hour in the PACU. A small difference appeared only from one hour in the PACU onwards (3-5 mmHg lower in the sugammadex group, p < 0.05), which is not clinically significant (Figure 2). From one hour after PACU admission onward, SBP was 3 mmHg to 5 mmHg lower in the sugammadex group. Although this difference was statistically significant (p < 0.05), it was not clinically relevant.

Tidal volume was recorded for 12 minutes following reversal as a measure of functional respiratory recovery (Table 4). Sugammadex produced a rapid rise in tidal volume, reaching 11.20 ± 4.56 mL/kg at five minutes compared with 8.03 ± 3.08 mL/kg with neostigmine (p < 0.001), with significant differences from two to nine minutes after reversal. Pre-reversal tidal volume was lower in the sugammadex group, but the difference was statistically insignificant. Peak tidal volume was 11.54 ± 4.63 in the sugammadex group versus 8.34 ± 3.10 mL/kg in neostigmine (p < 0.001). Every child in the sugammadex group achieved a tidal volume of at least 5 mL/kg, compared with 43 of 50 (86%) in the neostigmine group (p = 0.012). Higher tidal volumes in the early post-reversal period were associated with pediatric patients receiving sugammadex. These findings may suggest that faster neuromuscular recovery can translate into more effective spontaneous ventilation. The linear mixed-effects model demonstrated a statistically significant interaction between drug group and time for tidal volume/kg (χ² = 397.69, p < 0.001). Tidal volume/kg increased more rapidly over time in the sugammadex group than in the neostigmine group, indicating that the pattern of respiratory recovery differed significantly between the two groups.

**Table 4:** Tidal volume (mL/kg) following administration of the reversal agent (total n=100) Data are presented ias mean ± SD and compared by independent sample t-test. A linear mixed model analysis was used to present the interactions between drugs and tidal volume per kg over time (random-intercept variance =1.552, residual variance =0.440, χ² =397.69, p<0.001). A p<0.05 is considered significant. Peak tidal volume is the highest value recorded for each patient at any time point after administration of the reversal agent.

Tidal volume at various time points	Sugammadex group (mean ±SD)	Neostigmine group (mean ±SD)	Test statistics (t)	p-value^*^
Pre-reversal	6.65 ± 3.18	7.58 ± 3.04	1.495	0.138
One minute after reversal	8.24 ± 3.51	7.77 ± 3.06	0.714	0.858
Two minutes after reversal	9.82 ± 4.03	7.01 ± 2.80	4.049	<0.001
Three minutes after reversal	10.75 ± 4.24	8.17 ± 3.10	3.473	<0.001
Four minutes after reversal	11.19 ± 4.47	8.09 ± 3.11	4.025	<0.001
Five minutes after reversal	11.20 ± 4.56	8.03 ± 3.08	4.073	<0.001
Seven minutes after reversal	10.36 ± 4.22	7.88 ± 3.06	3.364	<0.001
Nine minutes after reversal	9.54 ± 3.96	7.80 ± 3.09	2.450	0.016
12 Minutes after reversal	8.67 ± 3.63	7.67 ± 2.99	1.504	0.220
Peak tidal volume achieved	11.54 ± 4.63	8.34 ± 3.10	4.061	<0.001

Apart from tachycardia in the neostigmine group, both reversal agents were well tolerated, with a low incidence of adverse events. Postoperative nausea and vomiting occurred in two patients in each group, while one patient in the neostigmine group developed hypersalivation. No episodes of bradycardia, hypotension, bronchospasm, or laryngospasm were observed in either group.

## Discussion

Our study demonstrated that sugammadex, in comparison to neostigmine, provided significantly faster reversal of vecuronium-induced neuromuscular blockade and less transient tachycardia in children aged two to 12 years undergoing elective surgery requiring general anesthesia. Rapid and complete reversal of neuromuscular blockade is particularly important in pediatric anesthesia because children have lower physiological reserve and are more susceptible to respiratory complications resulting from residual neuromuscular blockade [9]. In contrast to previous studies that primarily assessed neuromuscular recovery with limited evaluation of functional respiratory recovery, our study assessed tidal volume as a marker of respiratory function [10,11]. Higher early tidal volumes were observed with sugammadex and were associated with more rapid neuromuscular recovery; however, this secondary outcome was exploratory and does not establish a causal relationship between the two. 

The majority of published literature has evaluated the efficacy of sugammadex in reversing rocuronium-induced neuromuscular blockade, while evidence on its use for the reversal of vecuronium-induced blockade is limited [12-14]. To the best of the authors' knowledge, this is the first study from the Indian subcontinent to evaluate the efficacy of sugammadex for reversal of vecuronium-induced neuromuscular blockade in pediatric patients. Our findings are consistent with earlier studies of pediatric patients comparing sugammadex and neostigmine. The absolute recovery times are variable between studies; a structured comparison of study protocols and outcomes is provided in Table 5.

**Table 5:** Comparison of study protocols and recovery outcomes of sugammadex versus neostigmine for reversal of neuromuscular blockade in pediatric patients The values are presented as mean ± SD unless stated otherwise. NMBA: Neuromuscular blocking agent; T2: Second twitch of the train-of-four; TOF: Train-of-four; ASA: American Society of Anesthesiologists

Author names and year of publication	Population, age range, sample size	NMBA	Sugammadex dose	Comparison group	Depth of block at reversal	Monitoring modality	Recovery endpoint	Recovery time: Sugammadex vs comparative group
Present study (2026)	100 pediatric patients, two to 12 years, ASA grades I-III, elective surgery (50 per group)	Vecuronium	2 mg/kg	Neostigmine 50 µg/kg + glycopyrrolate 10 µg/kg	Moderate, reappearance of T2 (TOF count 2)	Acceleromyography, TOF 3D (MIPM device)	TOF ratio ≥0.9 and TOF ratio 1.0 (extubation criteria)	TOF 0.9: 1.2 vs 4.2 min (median 70 vs 252 sec); TOF 1.0: 1.4 vs 4.5 min (median 86 vs 272 sec)
Voss et al. (2025) [10]	138 treated, birth to <2 yr, ASA grades I-III; part B efficacy 29 vs 31	Rocuronium (71%) or vecuronium (29%)	2 mg/kg (moderate); 4 mg/kg (deep)	Neostigmine 50 µg/kg + glycopyrrolate 10 µg/kg or atropine 20 µg/kg	Moderate, return of T2; deep post-tetanic count 1-2	Quantitative neuromuscular transmission monitoring (TOF-watch sugammadex or peripheral nerve stimulator); 97.8% device-assessed; mostly accelerometry	Time to neuromuscular recovery (one of four methods, including TOF ≥0.9)	Part B: 1.4 vs 4.4 min hazard ratio 2.40 (1.37-4.18), p=0.0002; parts A+B: 1.2 vs 4.2 min deep, 4 mg/kg: 1.1 min
Özgün et al. (2014) [11]	60 children, 2-12 yr, ASA I-II, ENT surgery, supine (30 per group)	Rocuronium	2 mg/kg	Neostigmine 60 µg/kg + atropine 20 µg/kg	Moderate, reappearance of T2	Acceleromyography, TOF-watch SX; stabilization and baseline recorded before rocuronium (baseline TOF 97% to 100%)	Time to TOF ratio 0.9 (T0.9)	68 s (1.13 min) vs 392 sec (6.53 min), p<0.001; extubation: 202.5 vs 427.5 sec
Ammar et al. (2017) [12]	60 pediatric patients, two to 10 years, ASA grades I-II, lower abdominal surgery (30 in each group)	Rocuronium	4 mg/kg	Neostigmine 0.35 mg/kg + atropine 20 µg/kg (dose as printed)	Differed between groups: Sugammadex at post-tetanic coun 1-2 (deep); neostigmine at T2 (moderate)	Acceleromyography, TOF-watch; calibrated after induction before rocuronium; <60 mA, 0.2 ms, 2 Hz/15 s; bispectral index 45-55	Time to TOF ratio >0.9	2.5 ± 0.8 vs 12.6 ± 4.3 min (p=0.002); extubation: 2.0 ± 0.8 vs 4.3 ± 1.9 min; repeat dose needed: 1 vs 8 (p=0.035)
Plaud et al. (2009) [15]	Infants, children (two to 11 years), adolescents and adults; dose-finding	Rocuronium 0.6 mg/kg	0.5, 1, 2 or 4 mg/kg	Placebo - no neostigmine group; dose-finding design	Moderate, reappearance of T2	Quantitative TOF; propofol anesthesia at reversal	Time to TOF ratio ≥0.9	After 2 mg/kg: 0.6 (infants), 1.2 (children), 1.1 (adolescents), 1.2 min (adults)
Kara et al. (2014) [16]	80 pediatric patients, two to 12 years, ASA grade I, outpatient lower abdominal/urogenital surgery (40 per group)	Rocuronium	2 mg/kg	Neostigmine 30 µg/kg + atropine 10 µg/kg	Nominally T2, but TOF ratio at reversal differed: 28.6% (sugammadex) vs 47.3% (neostigmine), p=0.020	Acceleromyography, TOF-watch; calibrated to 100% after intubation; TOF not monitored during surgery	Time to TOF ratio >0.90	0.46 ± 0.70 vs 1.97 ± 2.14 min (p=0.002); extubation: 1.15 ± 1.44 vs 3.25 ± 1.79 min; TOF at extubation: 96.4% vs 77.0%
Ghoneim & El Beltagy (2015) [17]	40 pediatric patients, seven to 18 years, elective craniotomy for posterior fossa tumor	Rocuronium	4 mg/kg	Neostigmine 40 µg/kg + atropine 20 µg/kg	Moderate, TOF count 2	Quantitative TOF (after termination of sevoflurane)	Time to TOF ratio ≥0.9	1.4 ± 1.2 vs 25.16 ± 6.49 min
Saber et al. (2021) [18]	50 pediatric patients, <2 years, ASA grades II, III, congenital heart disease, cardiac catheterisation (25 in each group)	Rocuronium	4 mg/kg	Neostigmine 40 µg/kg + atropine 20 µg/kg	Moderate, reappearance of T2	Acceleromyography, TOF-watch sugammadex; calibrated and normalized before rocuronium	Time to TOF ratio 0.90	1.61 ± 0.50 vs 9.23 ± 3.71 min (p<0.001)

The majority of previous pediatric studies of sugammadex used rocuronium [11,12,15-18], except the study by Voss et al. [10], which included patients younger than two years, with approximately one-third receiving vecuronium. In our study, we used vecuronium, which has a longer duration of action and slower spontaneous recovery than rocuronium. The recovery times in our study also differed from those reported by Ozgün et al. and Kara et al. [11,16], highlighting the influence of study protocol on recovery estimates. The sugammadex recovery time in our study was very similar to that reported by Ozgün et al. (70 vs 68 seconds to TOF 0.9) [11], whereas recovery with neostigmine was faster in our study than in theirs (252 vs 392 seconds). Although both studies assessed recovery at reappearance of T2, the study protocol by Ozgün et al. [11] used rocuronium, neostigmine (60 mcg/kg), and atropine, while we used vecuronium, neostigmine (50 mcg/kg), and glycopyrrolate, which may have contributed to differences in recovery kinetics. In contrast, the substantially shorter recovery times reported by Kara et al. [16] require particular caution in interpretation. Although reversal was nominally administered at T2, the TOF ratio at reversal differed between their groups (28.6% with sugammadex vs 47.3% with neostigmine), and TOF was not monitored throughout surgery. These protocol differences make their recovery times less directly comparable with those of our study, in which both groups received reversal at a standardized and quantitatively confirmed reappearance of T2. Thus, differences in NMBA, depth of block, quantitative monitoring, and reversal protocol likely account for much of the variation in absolute recovery times across studies. Despite these differences in study protocols, all studies consistently showed faster recovery with sugammadex. 

The majority of previous pediatric studies of sugammadex used rocuronium [11,12,15-18], except the study by Voss et al. [10], which included patients younger than two years, with approximately one-third receiving vecuronium. In our study, we used vecuronium, which has a longer duration of action and slower spontaneous recovery than rocuronium. The recovery times in our study also differed from those reported by Ozgün et al. and Kara et al. [11,16], highlighting the influence of study protocol on recovery estimates. The sugammadex recovery time in our study was very similar to that reported by Ozgün et al. (70 vs 68 seconds to TOF 0.9) [11], whereas recovery with neostigmine was faster in our study than in theirs (252 vs 392 seconds). Although both studies assessed recovery at the reappearance of T2, the study protocol by Ozgün et al. [11] used rocuronium, neostigmine (60 mcg/kg), and atropine, while we used vecuronium, neostigmine (50 mcg/kg), and glycopyrrolate, which may have contributed to differences in recovery kinetics. In contrast, the substantially shorter recovery times reported by Kara et al. [16] require particular caution in interpretation. Although reversal was nominally administered at T2, the TOF ratio at reversal differed between their groups (28.6% with sugammadex vs 47.3% with neostigmine), and TOF was not monitored throughout surgery. These protocol differences make their recovery times less directly comparable with those of our study, in which both groups received reversal at a standardized and quantitatively confirmed reappearance of T2. Thus, differences in NMBA, depth of block, quantitative monitoring, and reversal protocol likely account for much of the variation in absolute recovery times across studies. Despite these differences in study protocols, all studies consistently showed faster recovery with sugammadex. 

Previous studies have reported tachycardia following neostigmine administration due to the glycopyrrolate component. Our study further provides a more comprehensive temporal assessment of hemodynamic parameters, showing that this effect extends into the early postoperative period [18]. Although rare cases of bradycardia and even asystole following sugammadex administration have been reported in the literature, these have predominantly occurred in patients with underlying cardiac disease [19-21]. We did not observe any such adverse events in our pediatric cohort.

A significant difference in postoperative respiratory complications between sugammadex and neostigmine has not been demonstrated [22]. Similarly, we observed no statistically significant difference between the groups, although our study was not powered to detect infrequent respiratory events. Although prospective pediatric data remain limited, sugammadex has been effectively used to reverse neuromuscular blockade in various clinical scenarios in children, including emergency 'cannot intubate, cannot ventilate' situations. Its safety has been established in children with neuromuscular disorders such as myasthenia gravis, Duchenne muscular dystrophy, and myotonic dystrophy [14].

A major strength of the study was the completeness of the data. It was a double-blind randomized trial. Randomization was performed towards the end of surgery, immediately before planned extubation, so that every exclusion decision preceded allocation, which removed the possibility of post-randomization exclusion bias and losses to follow-up. Reversal was administered after the reappearance of T2, making sure that all pediatric patients were reversed from a standard depth of neuromuscular blockade rather than at the clinician's discretion. The interval between the last vecuronium administration and reversal was also comparable between the two groups. The assessment of functional respiratory recovery by serial tidal volume measurements allowed demonstration of the clinical correlation between neuromuscular recovery and restoration of spontaneous ventilation. The two groups were comparable in age, sex, ASA status, BMI, duration of anesthesia, vecuronium dose, and the interval between the last dose of vecuronium and reversal. The analyzed sample of 50 pediatric patients per group exceeded the calculated requirement of 42.

The study has several limitations. We included children aged >2 years undergoing elective surgery; therefore, the findings may not apply to younger children or to those undergoing emergency surgical procedures. Although the observer was kept blinded to group allocation, the characteristic heart rate response to neostigmine may have permitted allocation to be inferred in some cases. But the primary outcome was measured by quantitative neuromuscular monitoring, so it is unlikely to be affected. The study was not powered to detect differences in infrequent respiratory complications. Only two such events occurred, both in the neostigmine group, but the difference between the two groups did not reach statistical significance.

The recently published 2025 European Society of Anaesthesiology and Intensive Care (ESAIC)/European Society for Paediatric Anaesthesiology (ESPA) guidelines on neuromuscular block in anesthetized children suggest the use of electromyography-based rather than acceleromyography-based quantitative monitoring [23]. It also recommends calibrating the monitor before administration of an NMBA. Our study employed acceleromyography (without pre-relaxant calibration), and guidelines caution that acceleromyography can yield baseline TOF ratios of 1.10 or higher, and a raw acceleromyographic TOF ratio of 0.9 may correspond to a normalized value below 0.9 and may therefore underestimate residual block. To mitigate this, tracheal extubation in our protocol was not performed at a TOF ratio of 0.9 but at a TOF ratio of 1.0, providing a safety margin above the conventional threshold. Quantitative monitoring was additionally continued for two hours in the PACU. Neuromuscular monitoring in awake pediatric patients has inherent practical difficulties, like a restless child displacing the thumb transducer. For this reason, monitoring was performed as intermittent measurements at predefined intervals rather than continuously. Since a similar monitoring protocol was applied to both groups, it is unlikely to have influenced the comparative data between the two groups presented in our study. The interval from discontinuation of sevoflurane to tracheal extubation was not recorded prospectively. However, sevoflurane was discontinued at a standardized point in the operative sequence in both groups (at the last surgical suture), so the depth of residual volatile anesthetic at the time of reversal is unlikely to have differed between the groups.

## Conclusions

This study was conducted to fill the existing gap in the literature, which has limited data to demonstrate the safety and efficacy of sugammadex in reversing vecuronium-induced muscle paralysis, especially in Indian pediatric patients. We found that sugammadex provides faster, clinically effective reversal of vecuronium-induced neuromuscular blockade and causes less tachycardia compared with neostigmine in children of two to 12 years of age. This makes it a valuable reversal agent in pediatric anesthetic practice, especially where rapid and reliable recovery is warranted.
